# Editorial: Behavioral addictions: Emerging science

**DOI:** 10.3389/fpsyt.2022.1127444

**Published:** 2023-01-10

**Authors:** Andreas Chatzittofis, Hyoun S. Kim

**Affiliations:** ^1^Medical School, University of Cyprus, Nicosia, Cyprus; ^2^Department of Clinical Sciences and Psychiatry, Umeå University, Umeå, Sweden; ^3^Department of Psychology, Toronto Metropolitan University (Formerly Ryerson University), Toronto, ON, Canada; ^4^University of Ottawa Institute of Mental Health Research at the Royal, Ottawa, ON, Canada

**Keywords:** behavioral addiction, shopping, COVID-19, gambling, sexual behaviors, internet use, gaming

## Introduction

In recent years, besides psychoactive substances, certain maladaptive behaviors have also been considered to be in the spectrum of addiction and classified as non-substance or behavioral addictions (1, 2). These behavioral addictions include those recognized in the International Statistical Classification of Diseases and Related health problems (11th ed.) (ICD-11) and the Diagnostic and Statistical Manual of Mental Disorders (DSM-5) such as gambling disorder and gaming disorder as well as putative behavioral addictions such as patterns of addictive internet use, problematic smartphone use, and shopping/buying disorder, while compulsive sexual behavior disorder is under “Impulse control disorder” (3, 4).

Although for some of these disorders there is preliminary data on the underlying mechanism, the pathophysiology is far from clear (5–7). As the debate on the exact phenomenology and classification of these mental health disorders is ongoing, there is an urgent need for further research in the field. In addition, the comorbidity of behavioral addictions with other psychiatric disorders is of importance as it raises new challenges for both the assessment and treatment of patients. The COVID-19 pandemic and the quarantine measures applied also had an effect on behavioral addictions with reported increases in the use of the internet, pornography, and gaming (8).

This Research Topic aims to gather new empirical data and highlight recent advances in behavioral addictions with a focus on comorbidity with other psychiatric disorders as well as the impact of the COVID-19 pandemic on behavioral addictions. We are excited to present the following articles, composing this Research Topic adding new elements to the understanding of these complex disorders, providing insight in their recognition and management.

## Understanding of behavioral addictions

Blinka et al. conducted a qualitative study of 23 men in treatment for problematic internet sex use focusing on the phenomenology of psychiatric symptoms. Common patterns were pornography use and cybersex, with continuous masturbation on a daily basis starting in early adulthood and continuing through the years. The symptoms were consisted with the addiction model with loss of control and pre-occupation being the most profound symptoms. Together with the onset of erectile dysfunction, negative consequences developed slowly and included life dissatisfaction, regret, and feelings of unfulfilled potential.

## Identification of risk factors and comorbidity

In a study of 325 healthy adults, Guo et al. applying network analysis, reported that the dimensions of impulsivity were closely associated with the components social media addiction and problematic smartphone use. The authors revealed that “motor impulsivity” was the most critical bridge node in both networks and propose it as a promising target for applying preventive and treatment interventions for social media addiction and problematic smartphone use.

Yun et al. performed a secondary analysis of the Panel Study on Korean Children reported an increase in problematic smartphone use between 2017 and 2018 with child's externalizing problems and permissive parenting behavior being the risk factors for preteens' problematic smartphone use. Protective factors included peer communication, parental supervision, and authoritative parenting behavior.

In another effort to identify personality traits as risk factors for problematic smartphone use in 184 children, Yoo et al., reported positive correlations between harm avoidance as personality trait and smartphone addiction proneness and addiction symptoms. In addition, the authors reported mediation effects of daily stress on the relationship between harm avoidance and Problematic Smartphone Use at baseline and follow up, suggesting that managing stress might be important in Problematic Smartphone Use with high harm avoidance.

Liu et al. investigated how the different components of the intolerance of uncertainty (IU) are associated with problematic smartphone use (PSU) *via* a symptom-level network approach. In a sample of 1,849 Chinese university students, the authors reported that the strongest pathway linking IU and PSU was between emotional reactions to uncertainty and coping-motivated smartphone use. Thus, the authors highlight the role of problematic smartphone use as a coping response to negative emotions derived from uncertainty and the potential of interventions targeting intolerance of uncertainty.

Regarding comorbidity, in a study by Machado et al., the authors investigated gender differences in adults seeking treatment for problematic internet use. Women had more psychiatric comorbidities compared to men and more severe behavioral addictions, such as compulsive buying and disordered eating. These women had also higher scores in impulsivity, novelty seeking, and self-transcendence compared to men. These results highlight the importance of assessing for co-occurring conditions in this clinical population.

Zhu et al. conducted a case-control study among 84 adolescents with adolescent non-suicidal self-injury to characterize the behavior addiction characteristics of the group. Factors such as being female, being only child, presence of internet addiction, and negative parenting styles were predictors of NSSI behavioral addiction characteristics in adolescents. Thus, the authors suggest that the development of coping strategies targeting this vulnerable group.

## The COVID 19 pandemic

Regarding the effects of the COVID 19 pandemic, Otis et al. investigated the gambling behavior of 85 sports gamblers during the course of the pandemic. The hypothesis on an initial decline in the early stages of the pandemic (due to availability restrictions), followed by an increase in gambling behaviors the months after the restart of live sporting events was partly supported, although gambling behaviors did not completely return to baseline levels. These results may have implications regarding legislation concerning access to gambling.

## Evidence based interventions

An extensive systematic review on the efficacy and tolerability of therapeutic interventions (psychological and pharmacological), for buying/shopping disorder is published by Vasiliu concluding that cognitive behavioral therapy (CBT) is supported by the current evidence, followed by the combination of CBT + antidepressants as well as monotherapy with serotoninergic antidepressants. This review helps clinicians to choose the most evidence-based treatment for these patients and emphasizes the need for high-quality trials.

In conclusion, recognizing the limitations of current knowledge, we should emphasize the need for further research in the field of behavioral addictions. Focus should be on the underlying mechanisms of these disorders elucidating both the phenomenology and pathophysiology helping the better classification and our understanding. Finally, research is urgently needed on applied interventions and management of behavioral addictions in order to minimize their burden on the population.

## Author contributions

All authors listed have made a substantial, direct, and intellectual contribution to the work and approved it for publication.
